# Evaluation of the Antifungal Effect of *Rhus verniciflua* Stokes Extract for Oral Application Potential

**DOI:** 10.3390/medicina59091642

**Published:** 2023-09-11

**Authors:** Yu-Rin Kim, Gyoo-Cheon Kim, Seoul-Hee Nam

**Affiliations:** 1 Department of Dental Hygiene, Silla University, Busan 46958, Republic of Korea; dbfls1712@hanmail.net; 2 Department of Oral Anatomy, School of Dentistry, Pusan National University, Yangsan 50612, Republic of Korea; 3 Department of Dental Hygiene, College of Health Science, Kangwon National University, Samcheok 25945, Republic of Korea

**Keywords:** *Candida albicans*, *Rhus verniciflua* Stokes, antifungal effect, antioxidant, oral health

## Abstract

*Background and Objectives*: This study confirms the possibility of using *Rhus verniciflua* Stokes (RVS) extract as a natural treatment for oral candidiasis. *Materials and Methods*: RVS was extracted with 70% ethanol to examine the antioxidant activity through polyphenol, flavonoid content, and DPPH (1,1-diphenyl-2-picrylhydrazyl). To evaluate the antifungal effect against *Candida albicans* (*C. albicans*; KCTC 7965/ATCC 10231) and evaluate the stability of RVS, a water-soluble tetrazolium salt (WST-1) assay was performed in human keratinocytes (HaCaT). *Results*: The findings revealed that RVS extract has fairly high antioxidant activity. The clear zones of the RVS extract against *C. albicans* increased in diameter due to the inhibition of fungal growth at higher concentrations. Treatment with the 1.25 mg/mL RVS extract had a more than 99% antifungal effect against *C. albicans*, and the 20 mg/mL RVS extract had a 100% antifungal effect. The WST-1 assay showed that the RVS extract induced low cell viability in the HaCaT cells, which inhibited their proliferation, and the RVS extract is also toxic to normal cells. *Conclusions*: Although the RVS extract with high antioxidant activity showed clear antifungal activity against *C. albicans*, it exhibited a low survival rate. Therefore, the development of a safe natural antibiotic is necessary.

## 1. Introduction

The oral cavity is an important organ in the human body, exposed to many microorganisms because it communicates directly with the outside world and offers environmental conditions suitable for the growth of bacteria, resulting in various oral diseases [1]. Moreover, as the importance of oral health has increased, especially due to the rise in mask-wearing as a result of viral infections, interest in managing bacteria in the mouth has increased [2]. Candida accounts for 70–90% of fungal infections that can occur in humans and can have fatal consequences when acquired in intensive care units, with a mortality rate of 40% [3]. In cases of long-term use of antibiotics or reduced immunity, intra-oral resident bacteria cause opportunistic infections. Among them, oral candidiasis occurs when yeast-type fungi of the genus *Candida* overgrow in the oral cavity [4]. Types of *Candida* include *C. albicans*, *C. tropicalis*, *C. glabrata*, *C. pseudotropicalis*, *C. guillierimondii*, *C. krusei*, *C. lusitaniae*, *C. parapsilosis*, and *C. stellatoidea* [4]. More than 80% of *Candida* are isolated as *C. albicans*, *C. glabrata*, and *C. tropicalis* [5]. *C. albicans* usually exists in the oral cavity and normally does not cause problems in healthy people [4]. In ordinary people, the carriage rate without symptoms is known to be 20–75% [6], but the incidence rate of oral candidiasis induced by *C. albicans* has been reported to be 30–45% in healthy adults [7]. The incidence rate of oral candidiasis caused by *C. albicans* isolated from the oral cavity is 54% in two- to six-week-olds, 46% for one-year-olds, 39% for one- to six-year-olds [8], and 30–45% in healthy adults [7]. In recent years, higher incidences of the above-mentioned non-*C. albicans Candida* (NCAC) species have also been reported [9]. In particular, for all cancer treatments, the weighted prevalence of oral colonization with fungal organisms was 48.2% before treatment, 72.2% during treatment, and 70.1% after treatment [10,11].

The incidence of oral candidiasis was 50–65% in patients using removable dentures and 65–88% in patients receiving long-term treatment for acute diseases [7,12]. Local factors that induce candidiasis include denture use, salivary malfunction, steroid inhalation, and oral cancer. The most common contributing factors include age, smoking, diabetes, Cushing’s syndrome, decreased immunity, malignant tumors, nutritional deficiencies, and antibiotic use [13]. Symptoms of oral candidiasis cause problems with oral tissues, such as the mucous membrane and tongue in the oral cavity, leading to discomfort, burning pain, dysgeusia, and dysphagia during chewing [14]. The first line of defense against oral candidiasis infection is innate immunity, which relies on immune cells (e.g., macrophages, dendritic cells, neutrophils, natural killer cells, monocytes, etc.) to defend against invasion [15]. During fungal infection, innate immune cells can recognize fungal PAMPs via PRRs to inhibit, phagocytose, and kill the fungus while initiating the innate immune response against pathogens [16]. Oral candidiasis can be naturally cured if the causative factor can be removed, and innate immunity can be enhanced. However, in most cases, antifungal treatment is required. Representative drugs for antifungal treatment of oral candidiasis are polyene-based and azole-based drugs [17]. Polyene-based drugs are not absorbed into the gastrointestinal tract, so they are either applied to the oral mucosa or taken orally. However, these drugs are difficult to use due to the discomfort of frequently applying them and their unpleasant taste. In many cases, the drugs are not sufficiently applied to the lesion because of obstructing structures in the oral cavity, such as the tongue, palate, gum, and teeth, or because they are diluted by the saliva of the patients [17]. Moreover, natural ingredients are in the spotlight because they have fewer side effects than chemical substances and, unlike chemical substances, do not cause resistance, mutation, and cytotoxicity [18]. Promising antifungal agents extracted from natural compounds isolated from plants are currently attracting much attention as potential cures for various diseases and as sources of new drugs [19]. Therefore, studies are being actively conducted to develop natural products or food-derived natural antifungal agents that outperform synthetic antifungal agents in terms of safety and effectiveness [20]. Medicinal plants from orders such as *Acorales* Mart., *Apiales* Nakai, *Asterales* Link, *Lamiales* Bromhead, *La-urales* Juss. ex Bercht. and J. Presl, *Myrtales* Juss. ex Bercht. and J. Presl, *Poales* Small, and *Sapindales* Juss. ex Bercht. and J. Presl have exhibited strong anti-biofilm activity against *Candida* species [21]. Among them, *Rhus verniciflua* Stokes (RVS) is an Asian tree species in the Anacardiaceae family. It is used in East Asian countries such as Korea, Japan, and China as a traditional herbal medicine for gastrointestinal diseases and pain relief in various diseases such as cancer [22]. RVS contains several compounds, such as quercetin, fustin, fisetin, sulferetin, and butane [23], which are reported to have antioxidant, anti-proliferative, anti-inflammatory, and anti-tumor effects [24]. Specifically, RVS has anti-tumor effects in the stomach, breast, liver, lymph nodes, and bones [25]. The mechanism underlying the anti-tumor effect of RVS is known as the activation of AMP-activated protein kinase (AMPK) [26], cell cycle arrest [27], activation of manganese superoxidase or reduction in glutathione content [28], and activation of caspase [16]. Despite the pharmacological effect of RVS, its use is very limited because it contains an allergen called urushiol. Various detoxification methods for RVS have been developed to remove its urushiol content [29]. However, since continuous use of natural drugs may expose patients to potential dangers, they should be carefully applied after ensuring their stability [30]. Posadzki et al. [31] reported that 19 out of 50 natural drugs had moderate or severe adverse events. Other studies claimed that hepatotoxicity or renal toxicity occurred frequently due to the toxic effects of natural drugs [32]. The potential risks of using natural drugs can arise from their contamination, incorporation, misrecognition, interaction with other drugs, and inherent toxicity. Therefore, for the safe use of natural drugs, they must undergo safety evaluation and quality control [33]. This study was conducted to confirm the possibility of applying a natural plant with an antioxidant effect and evaluate the antifungal effect(s) of RVS extract against *C. albicans* and its effect on cell viability. This study also provides data on animal experiments to confirm the potential of RVS as an antifungal agent for patients with oral candidiasis.

## 2. Materials and Methods

### 2.1. Extract Preparation

RVS-producing areas are located in Gyeongsan, Gyeongsangbuk, Republic of Korea, and were purchased from Cheongmyeong Co., Ltd. (Chungju, Republic of Korea). Ethanol extract was obtained via a rotary vacuum evaporator (N-1300E.V.S., Tokyo Rikakikai Co., Ltd. (EYELA), Tokyo, Japan) after filtration by adding 70% ethanol to the pulverized RVS at 60 °C for 12 h. RVS powder samples were obtained by freeze-drying through dehydration.

### 2.2. Assay for Total Polyphenol Content

The total polyphenol content was determined using the modified Folin-Denis method [34]. This involved adding 0.15 mL of Foline-Ciocalteau’s phenol reagent (Sigma-Aldrich, St. Louis, MO, USA) and 0.3 mL of 20% Na_2_CO_3_ (Daejung chemicals & Metals Co., LTD., Gyeonggi, Republic of Korea) into 1 mg/mL RVS extract. The mixture was left to react for 2 h in a dark room at room temperature, after which the absorbance was measured at 725 nm using a spectrophotometer (Thermo Scientific Multiskan GO, Thermo Fisher Scientific, Waltham, MA, USA). Gallic acid (Sigma-Aldrich Co., St. Louis, MO, USA) was used as a reference material to calculate the total polyphenol content of RVS extract and was expressed in terms of gallic acid equivalents (mg GAE/g extract). Average values were calculated from the triplicate experiment.

### 2.3. Assay for Total Flavonoid Content

The total flavonoid content was determined using the modified Davis WB’s method [35]. This involved mixing 0.7 mL of diethylene glycol with 0.1 mL of RVS extract and adding 0.1 mL of 1 N NaOH solution. The mixture was left to react for 1 h in a dark room at room temperature, after which the absorbance was measured at 420 nm using a spectrophotometer (Thermo Scientific Multiskan GO, Thermo Fisher Scientific, Waltham, MA, USA). Catechin (Sigma-Aldrich Co., St. Louis, MO, USA) was used as a reference material to calculate the total flavonoid content of RVS extract, expressed in terms of catechin equivalents (mg CE/g extract). Average values were calculated from triplicate experiments.

### 2.4. Measurement of DPPH Radical Scavenging Activity

DPPH (1,1-diphenyl-2-picrylhydrazyl, Sigma Chemical Co., St. Louis, MO, USA) radical scavenging activity was evaluated using the modified Sharma and Bhat’s method [36]. This involved adding 0.2 mL of RVS extract to 0.8 mL of 0.2 mM DPPH solution. The mixture was left to react in a dark room for 30 min, after which the absorbance was measured at 517 nm using a spectrophotometer (Thermo Scientific Multiskan GO, Thermo Fisher Scientific, Waltham, MA, USA). Sterile distilled water was added as a control instead of the sample, gallic acid was used as a positive control, and the radical scavenging activity was expressed as a percentage (%). The antioxidant activity was expressed by obtaining the electron-donating ability (%) using the formula [1 − (sample absorbance/control absorbance)] × 100. Average values were calculated from the triplicate experiment.

### 2.5. Fungal Cultures

The *C. albicans* (KCTC 7965/ATCC 10231) were incubated in yeast mold broth (YM, Difco, MI, USA) and cultivated at 37 °C for 24 h. Then, it was inoculated with the *C. albicans* cultured with 1 × 10^5^ colony-forming units per milliliter (CFU/mL).

### 2.6. Disc Diffusion Method

To confirm the antifungal effect of RVS, 100 μL of each concentration was dropped onto a paper disc (Ø8 mm; Advantec Toyo Kaisha, Ltd., Tokyo, Japan) of YM agar medium and aerobically incubated at 37 °C for 24 h. The diameter of the clear zone around was measured with the soaked filter paper discs using a ruler. Experiments were performed in triplicate.

### 2.7. Antifungal Activity

To measure the fungal proliferation, the extracts were prepared with RSV concentrations of 1.25, 2.5, 5, 10, and 20 mg/mL, which were inoculated with a 100 µL solution with *C. albicans*. To use an equal amount of *C. albicans*, the culture absorbance was maintained at 1.0 at a wavelength of 660 nm. The mixture was cultured aerobically at 37 °C for 24 h and diluted. A precise amount of it was spread onto a YM agar medium. The CFU was counted after incubation for 24 h, and the results of all colonies appearing on the YM agar medium were recorded. The data were based on triplicated experiments.

### 2.8. Cell Proliferation Assay

A water-soluble tetrazolium salt (WST-1) assay was performed [37] to quantify the effect of RVS on cell growth. HaCaT cells used in this experiment were purchased from the Department of Oral Anatomy, Pusan National University School of Dentistry (Yangsan, Republic of Korea). HaCaT cells were cultured in Dulbecco’s Modified Eagle’s Medium (DMEM) (USA), with 10% (*v*/*v*) heat-inactivated fetal bovine serum (Gibco, Grand Island, NY, USA) and 1% penicillin/streptomycin (Gibco, Grand Island, NY, USA) at 37 °C and with 5% CO_2_ in a humidified chamber. The experiments were performed in 96-well plates that contained a final volume of 100 μL of the medium/well. The HaCaT cells were seeded at an initial density of 1 × 10^5^ cells/cm^2^ and incubated at 37 °C for 24 h. The medium was changed to serum-free DMEM that contained the indicated concentrations of RVS (1.25, 2.5, 5, 10, and 20 mg/mL). After 3 h, the incubation medium was removed and replaced with the WST-1 solution. The plates were incubated at 37 °C for 2 h, and an ELISA reader (Multiskan FC, Thermo Fisher Scientific, Waltham, MA, USA) was used to measure the 450 nm absorption. The cell viability was confirmed by optical density, and the same process was repeated thrice.

### 2.9. Statistical Analysis

Statistical analysis was performed using a statistical software (SPSS v. 24.0, SPSS Inc., Chicago, IL, USA) to evaluate the antifungal activity of RVS through one-way ANOVA and Duncan’s test at a significance level of 0.05.

## 3. Results

### 3.1. Antioxidant Activity Assays

Analysis of the total polyphenol content, total flavonoid content, and DPPH radical scavenging activity at RVS extract 1 mg/mL concentration showed that the total polyphenol content was 501.405 mg GAE/g, the total flavonoid content was 1634.153 mg CE/g, and the DPPH radical scavenging activity was 72.228% (Table 1).

### 3.2. Antifungal Activity and Growth Inhibition of C. albicans

**Figure 1:**
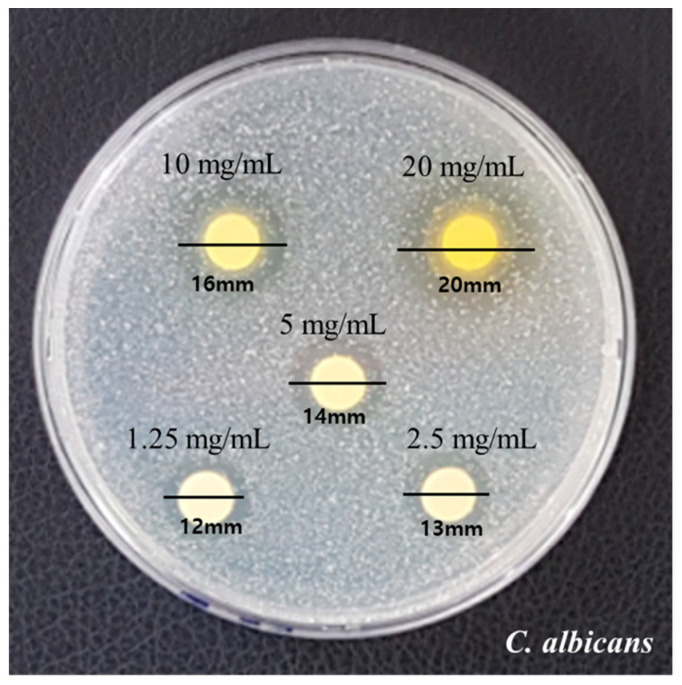
Results of the clear zone of RVS extract against *C. albicans*.

The measured diameters by the disk diffusion method are presented in Figure 1. Growth inhibition showed clear zones with diameters 12 mm, 13 mm, and 14 mm at concentrations of 1.25 mg/mL, 2.5 mg/mL, and 5 mg/mL, respectively. Diameters of 16 mm and 20 mm were observed at 10 mg/mL and 20 mg/mL, respectively. The fungal growth and inhibition area increased as the concentration of RVS extract increased.

**Figure 2:**
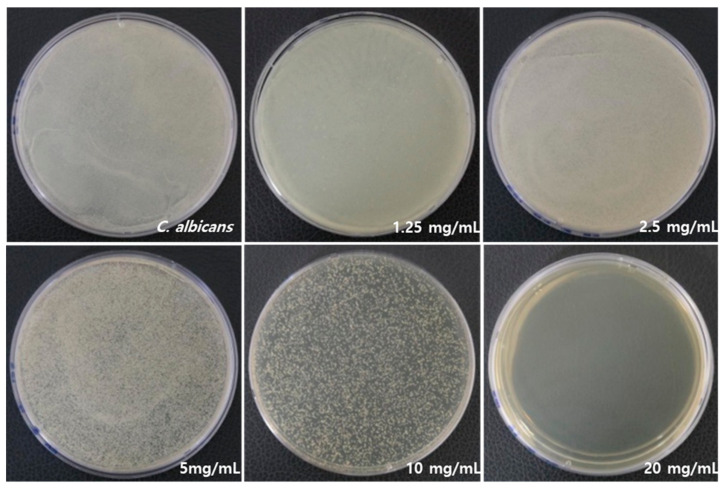
Inhibitory effects on the growth of *C. albicans* by RVS extract at concentrations of 1.25, 2.5, 5, 10, and 20 mg/mL.

Figure 2 illustrates a significant difference in antifungal activity against *C. albicans*. RVS extract had an antifungal effect, and no fungal growth was observed at 20 mg/mL. Table 2 lists the mean and standard deviation (SD) values of *C. albicans* count, which were 2.5 ± 0.1 109. As the concentration of RVS extract increased, higher growth inhibition was observed. The minimal fungicidal concentration (MFC) was determined to be 1.25 mg/mL of RVS extract, and 99% of the fungi were killed compared to the control *C. albicans* as a result of CFU.

### 3.3. RVS Extract on the Growth of HaCaT Cells

**Figure 3:**
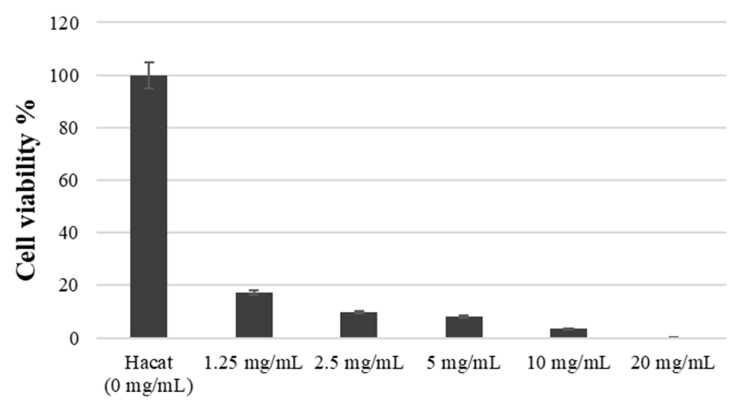
Cell viability at various treatment concentrations of RVS extract by the WST-1 assay.

The number of viable cells by RVS extract was assessed using WST-1 assay. Figure 3 demonstrates the analysis results, with the absorbance value of the control group set at 100%. After exposure to concentrations of 1.25 mg/mL, 2.5 mg/mL, 5 mg/mL, 10 mg/mL, and 20 mg/mL for 3 h, cell viability was measured as 17.21 ± 2.02, 9.87 ± 3.01, 7.96 ± 2.10, 3.35 ± 1.73, 0.15 ± 0.05, respectively (*p* < 0.05). As depicted in Figure 3, the RVS extract induced HaCaT cell death at concentrations ranging from 1.25 to 20 mg/mL, indicating a significant reduction in cell viability.

## 4. Discussion

Oral health is a multifaceted aspect of well-being that affects individuals physically, mentally, and socially. It needs to be approached as a part of general health and is an essential element in social and daily life [38]. The increase in life expectancy has led to a growing interest in quality of life, particularly oral health, which is closely related to dietary habits [39]. Therefore, effective oral health management is essential to prevent oral diseases and address their underlying causes [40].

*C. albicans* is an opportunistic fungus present in the human oral cavity and can be converted into disease-causing hyphae under nutrient-rich conditions [41]. *C. albicans* has an organic relationship with *S. aureus*, which strengthens bacterial colonization and biofilm formation, providing a scaffold for bacterial biofilm development. Thus, *C. albicans* survives even after antibiotic treatment and infects or destroys mucosal tissues [42]. It is eliminated with topical antifungal treatments. However, Nystatin oral solution and clotrimazole tablets have high concentrations of sugar, which may cause dental caries [43]. Fluconazole or itraconazole is known to be effective when patients are at risk of systemic fungal infections or do not respond to topical drug treatments. Nevertheless, these drugs have clinical limitations due to their discomfort, unpleasant taste, and the risk of developing azole-resistant strains when used repeatedly [44]. Therefore, research on alternative products continues, considering that natural substances isolated from plants used in traditional medicine are good alternatives to synthetic chemicals. Interest in adverse substances with environmentally friendly functions is increasing [45]. Recently, studies on disease treatment and prevention using natural products and the development of health-functional materials have been actively conducted [46]. Therefore, the viability of natural antioxidants is evaluated by measuring antioxidant compounds and their activity. Various physiological activities, such as antioxidant, antifungal, and anti-inflammatory properties, of phenolic compounds and flavonoids present in large amounts in plants are evaluated [47]. Phenolic compounds, which are secondary metabolites widely distributed in nature, typically exhibit physiological activities such as antioxidant effects as the total polyphenol content increases [48]. In addition, flavonoids, which are mainly present in plants, are highly active antioxidants and are known to have antiviral, anti-inflammatory, and anticancer effects similar to polyphenols [49]. DPPH is a substance with chemically stabilized free radicals and is reduced by substances such as ascorbic acid, tocopherol, polyhydroxy aromatic compounds, leading to the decolorization of its dark purple color. This is an extensively used method to measure antioxidant activity by determining the electron-donating ability of antioxidants derived from various natural materials [50]. The results of analyzing the antioxidant activity of RVS extract in this study reveal that the total polyphenol content was 501.405 mg GAE/g, the total flavonoid content was 1634.153 mg CE/g, and the DPPH radical scavenging activity was 72.228%. These values indicate a similar activity to the 78.508% activity of the control substance, gallic acid. According to Sim WS et al. [51], who studied the antioxidant activity of nine domestic forest plants, *Geranium thunbergii* showed the highest total polyphenol content at 303.94 ± 0.63 mg GAE/g, while *Vitis ficifolia* was found to have the highest flavonoid content at 279.00 ± 4.58 mg CE/g. These results indicate that RVS extract has a high polyphenol and flavonoid content. Although the polyphenol content and flavonoid content of natural products differ depending on their type, they generally exhibit anti-inflammatory properties. In particular, the RVS extract used in this study is a natural substance known for improving the oral health and has excellent anti-inflammatory properties. Therefore, these results are promising, suggesting the possibility of its application in oral health products. In addition, compared to a study [52] reporting that *Osmunda japonica’s* DPPH scavenging activity was 67.29%, the RVS extract demonstrated remarkably high radical scavenging activity. The RVS extract was also confirmed to have excellent antioxidant activity by effectively inhibiting the presence of reactive oxygen species. The RVS extract will be able to suppress body aging by activating the antioxidant defense system or scavenging active oxygen. This study confirmed the antifungal effect of RVS extract against *C. albicans*. The observed zone size in the disk diffusion test indicated that the higher the concentration of the RVS extract, the wider the clear zone. The antifungal activity of RVS extract exhibited more than 99% inhibition rate at a concentration of 1.25 mg/mL. Similar to the results of this study, Shin et al. [53] used *Rubus coreanus Miquel* and reported that it had a strong growth inhibitory effect of over 90% at 60 mg/mL. In addition, Choi et al. [54] reported that *C. albicans* was not detected at 50 mg/mL when applying *Acanthopanax sessiliflorum* extract. While the natural extract used in their study is excellent for inhibiting the growth of C. albicans, the RVS extract used in this study, even at lower concentrations, was effective in killing C. albicans.

Nevertheless, the peel of RVS extract mainly contains urushiol, an allergic compound, which limits its medical use [55]. HaCaT cells are derived from adult skin and are commonly used in scientific research as oral epithelial cells [56]. In this study, verifying the low cell viability using HaCaT cells was necessary to identify the effect of specific fungi using natural materials. Therefore, the application of RVS to HaCaT cells confirmed a significantly reduced survival rate. Consequently, while the RVS extract exhibited excellent antifungal properties against *C. albicans*, it also led to a decrease in cell viability.

*Chelerythrine*, extracted from Bocconia Houttuynia cordata Thunb, is an effective natural drug against *C. albicans* and possesses various properties, such as anti-cancer, anti-inflammatory, insecticide, and anti-fibrotic activities [57]. In addition, several other natural drugs, such as *Perilla frutescence*, *Radix pulsatilla* (Bai Tou Weng), *Cortex phellodendri* (Huang Bai), *Rhizoma coptidis* (Huang Lian), *Cortex fraxini* (Qin Pi), *Coptis chinensis Franch*, *Phellodendron chinense C.K.Schneid*, *Paeonia suffruticosa*, *Paeonia Suffruticoas Andr*, *Magnolia officinalis*, *Dioscorea nipponica Makino*, and *Houttuynia cordata Thunb* (Saururaceae family), are involved in innate immunity against *C. albicans* [58]. As such, studies on the antifungal efficacy of various natural drugs effective against *C. albicans* are being actively conducted, but studies on their cytotoxicity should also be conducted to ensure their safety. Therefore, this study is valuable because it confirmed the antioxidant activity of RVS extract, the growth inhibition effect against *C. albicans*, and the low survival rate against HaCaT cells.

The current study’s findings provide fundamental research data and suggest the possibility of developing antioxidant materials. However, this study has limitations. Firstly, anti-Candida properties were not confirmed using various methods. Therefore, further studies are needed to confirm the effect of RVS on biofilm formation and the morphological conversion of *C. albicans*. In addition, investigating reactive oxygen species (ROS) production, adenosine triphosphate (ATP) consumption, RNA extraction, and quantitative RT-PCR analysis is required to identify the mechanism underlying the potential antifungal effect. Secondly, the safety of the RVS extract was not secured using more diverse normal cells. Accordingly, it is essential to identify various cell types, such as human normal oral keratinocytes, human gingival fibroblasts, and B16 F10 mouse melanoma cells, in cytotoxicity experiments to ensure safety. Thirdly, comparing the results of this study with existing studies should be done cautiously because there is a difference in the experimental method. Therefore, analyzing the inhibitory effect of *C. albicans* by extending the period to 48, 72, and 24 h and exploring various RVS extraction methods are needed to determine the most effective extraction method. Fourthly, since this study is conducted in a laboratory setting using bacterial cultures, it is difficult to generalize the research results because the human oral environment and conditions are not the same. Consequently, before RVS extract is used to treat oral candidiasis, its efficacy as a natural antifungal agent, and its non-toxicity, it is crucial to conduct extensive animal studies. In addition, after the safety of RVS is secured, a clinical study on the risk of recurrence of fungal infection should be conducted with an extended observation period in the future. The potential usability of RVS extract in the pharmaceutical industry can be realized through further research on its functionality and safety.

## 5. Conclusions

The study’s results confirmed that RVS extract had a more than 99% antifungal effect against *C. albicans* at a concentration of 1.25 mg/mL. Moreover, HaCaT cells, which are oral epithelial cells, showed cytotoxicity, and only 17.21% of them survived at a concentration of 1.25 mg/mL. Therefore, while RVS extract inhibits the growth of *C. albicans*, the survival rate of HaCaT cells is low. Hence, further research is needed to identify its appropriate concentration for its safe and effective use.

## Figures and Tables

**Table 1:** Total phenolic content (mg GAE/g extract), flavonoid content (mg CE/g extract), and DPPH radical scavenging activity (%) of RVS extract. Values are the mean ± SD, which were triplicated (*n* = 3).

Sample	Total Polyphenol Content	Total Flavonoid Content	DPPH Radical Scavenging Activity
RVS extract	501.405 ± 17.037	1634.153 ± 18.314	72.228 ± 0.450

**Table 2:** Mean ± S.D CFU values for the antifungal effect of *C. albicans* according to the RVS extract concentration (Unit: CFU/mL).

*C. albicans*	1.25 mg/mL	2.5 mg/mL	5 mg/mL	10 mg/mL	20 mg/mL	ANOVA *p*-Value
2.5 ± 0.1 10^9 a^	3.8 ± 0.3 10^6 b^	2.0 ± 0.2 10^6 c^	1.5 ± 0.1 10^4 d^	4.2 ± 0.2 10^3 e^	0.0 ^e^	0.000 *

* The significant difference among groups in one-way ANOVA. Different letters (a, b, c, d, and e) represent significant results in the post hoc Duncan test (*p* < 0.05).

## Data Availability

Not applicable.
